# Immune response to *Propionibacterium acnes* in patients with sarcoidosis – in vivo and in vitro

**DOI:** 10.1186/s12890-015-0070-7

**Published:** 2015-07-24

**Authors:** Jonas Christian Schupp, Sandrine Tchaptchet, Niklas Lützen, Peggy Engelhard, Joachim Müller-Quernheim, Marina A Freudenberg, Antje Prasse

**Affiliations:** Department of Pneumology, University Medical Centre, Albert-Ludwigs University, Killianstr. 5, 79106 Freiburg, Germany; Department of Developmental Immunology, Max Planck Institute of Immunobiology und Epigenetics, Freiburg, Germany; Department of Radiology, University Medical Centre, Freiburg, Germany; Faculty of Biology, University of Freiburg, Freiburg, Germany; Department of Pneumology, Medical School, Hannover, Germany

**Keywords:** Sarcoidosis, human, *Propionibacterium acnes*, Immunoglobulin, Immune response

## Abstract

**Background:**

*Propionibacterium acnes* was found in lungs and lymph nodes of patients with sarcoidosis and may induce hypersensitivity type granuloma formation. Data regarding the immune response to *P. acnes* of European sarcoid patients are scarce.

**Methods:**

We assessed the total IgG and IgA amount and specific antibodies to *P. acnes* and to *Staphylococcus aureus*, serving as a control, in BAL fluid of 64 patients with sarcoidosis and of 21 healthy volunteers. In a subcohort of sarcoid patients and controls, TNF-α and GM-CSF production of BAL cells stimulated with heat-killed *P. acnes* were measured.

**Results:**

In sarcoid patients, the total IgG and IgA levels in BAL fluid were significantly elevated compared to healthy volunteers. IgG and IgA titres against *P. acnes* and *S. aureus* were increased in sarcoid patients, yet based on the total amount of antibodies, only antibodies directed against *P. acnes* were relatively and significantly increased. Furthermore, BAL cells of sarcoid patients produced significantly more TNF-α and GM-CSF upon stimulation with heat-killed *P. acnes* compared to controls.

**Conclusions:**

Patients with sarcoidosis had elevated levels of specific antibodies against *P. acnes* which suggest contact with this bacterium in the past. Furthermore, BAL cells of sarcoid patients produced inflammatory cytokines (TNF-α and GM-CSF) upon stimulation with *P. acnes* indicating potential involvement of this pathogen in the pathogenesis of sarcoidosis in some patients.

## Background

Sarcoidosis is a systemic, granulomatous, and non-infectious disease, which most often affects the lungs. The key feature of sarcoidosis is a strong interaction of T-cells and macrophages leading to an exaggerated T_H_1 immune response, which is thought to be triggered by antigens in susceptible individuals. The obvious clinical heterogeneity of sarcoidosis supports the concept that several different sources of antigens such as bacteria, foreign particles, autoantigens or other endogenous factors may be involved. In the past decade, considerable progress has been made in defining potential antigens involved in sarcoidosis [1–4]. Especially antigens either derived from mycobacteria or from propionibacteria have been associated with sarcoidosis. For both pathogens a specific T-cell response was demonstrated in a high percentage of sarcoid patients [5–9].

Propionibacteria are gram-positive and aero-tolerant anaerobic bacteria. Particularly *Propionibacterium acnes (P. acnes*) is part of the skin, large intestine, mouth and conjunctiva flora and a common resident of the lung [10]. Mycobacteria and propionibacteria share many common features. Most importantly, both are intracellular pathogens and known for their persistence in macrophages due to the extraordinary high lipid content of their cell walls [11].

In the 80s of the last century *P. acnes* was identified as a putative causative agent of sarcoidosis [3]. The group of Abe and Homma cultivated *P. acnes* in homogenates of lymph nodes of patients with sarcoidosis [12]. Since then, *P. acnes* was detected by several, more sophisticated, methods: Hiramatsu et al. found elevated propionibacterial DNA in bronchoalveolar lavage cells from patients with sarcoidosis compared to healthy volunteers [13], Ishige and Ichikawa et al. confirmed and quantified the propionibacterial DNA in BAL cells, lymph nodes and lungs tissue [10, 14] and Negi et al. detected *P. acnes* antigens with monoclonal antibodies in tissue sections [15] of sarcoid patients. Significantly higher levels of *P. acnes* genome were reported in sarcoid patients compared to patients with tuberculosis or healthy controls [16]. In addition, antibodies directed against RP35, a fragment of the *P. acnes* trigger factor, were found to be elevated in BAL fluid of sarcoidosis patients [17]. Most of these data are derived from studies by Japanese groups testing Japanese patients, while only very limited data exist for Caucasian patients. Furthermore, *P. acnes* is able to provoke a granulomatous T_H_1 inflammation in mice mimicking sarcoidosis [18–21]. Based on the Japanese data, Y. Eishi formulated the hypothesis that, in sarcoid patients, a latent *P. acnes* lung and/or lymph node infection can lead to a hypersensitivity granuloma formation directed against *P. acnes* and it’s derived proteins [3].

In sarcoidosis, elevation of several proinflammatory cytokines has been shown [22]. Tumour necrosis factor alpha (TNF-α) and granulocyte macrophage colony-stimulating factor (GM-CSF) are two key cytokines: TNF-α is required for granuloma formation, as shown in several studies in mice [23, 24] and GM-CSF is able to induce transformation of alveolar macrophages into multinucleated giant cells [25]. Both, granulomas and multinucleated giant cells, are the hallmark histopathological changes in sarcoidosis.

Based on these data we investigated whether specific *P. acnes* directed immunoglobulin production could be detected in Caucasian sarcoid patients, as specific antibodies reflect a previous, *in vivo*, immune response to *P. acnes*. As well, we attempted to prove an immunologic response of BAL cells to *P. acnes in vitro*.

## Methods

### Subjects

64 patients with sarcoidosis who underwent bronchoscopy for routine diagnostic evaluation and 21 healthy volunteers were included after obtaining their written informed consent. Diagnosis was made by clinical evaluation, HRCT, laboratory and histologic findings (non-caseating epitheloid granulomas on tissue biopsies) according to the American Thoracic Society/European Respiratory Society/World Association for Sarcoidosis and Other Granulomatous Disorders (ATS/ERS/WASOG) statement. Bronchoscopy and bronchoalveolar lavage (BAL) was performed using standard technique [26]. BAL cells were processed as previously described [26]. The study was approved by the local ethics committee of Albert-Ludwig University Freiburg (231/03).

### IgG- and IgA-ELISA

We performed the following ELISA to quantify the total amount of IgG and IgA: 96 well plates were coated with 1:1000 diluted goat anti-human IgG + IgM + IgA antibodies (Dianova, Hamburg, Germany). We used 1:300 diluted goat anti-human IgG / IgA conjugated with alkaline phosphatase (Dianova, Hamburg, Germany) as detection antibodies and p-nitrophenylphosphate (Sigma-Aldrich, St. Louis, USA) as substrate. The detection limits were 0.8 ng/ml total IgG and total IgA, respectively.

### Preparation of *P. acnes* or *S. aureus* antigen

*Propionibacterium acnes* (ATCC 12930) and *Staphylococcus aureus* (*S. aureus*) were cultured, heat-killed and lyophilised as previously described [27]. The lyophilisate was resuspended in carbonate buffer (8.4 g NaHCO_3_ & 3.56 g Na_2_CO_3_ in 1 l H_2_O, pH 9.5) by sonification (at 4 °C for 30 min). The bacterial suspension was centrifuged (at room temperature for 2 min, 20000 g) and concentration of the supernatant (antigen) was measured using BCA protein assay (Pierce, Bonn) and adjusted with carbonate buffer to 1 mg/ml. *P. acnes* and *S. aureus* antigens were used for specific ELISA and stimulation of BAL cells.

### Anti-*P. acnes*-IgG/IgA- and Anti-*S. aureus*-IgG/IgA-ELISA

To measure the relative specific antibodies against *P. acnes* and *S. aureus* the following ELISA was utilized: 1 μg *P. acnes* or 0.7 μg *S. aureus* bacterial antigen was coated on 96-well ELISA maxisorb plates (Nunc, Roskilde, Denmark) and incubated at 37 °C for one hour followed by 4 °C over-night. The plates were washed twice with washing buffer (0.1 % Tween 20 in PBS), then blocked with 200 μl blocking buffer (2 % BSA in PBS) at room temperature for one hour. The BAL fluids were diluted with blocking buffer; one serum was used as standard in each experiment. After washing, the plates were loaded with standard or samples in triplicates, incubated at room temperature for two hours and then washed again. As detection antibodies we used 25 ng alkaline phosphatase linked anti-human IgA or IgA (Southern Biotechnology, USA) per well. 50 μg pNPP (Sigma, St. Louis, USA) per well served as substrate. Optical density (OD) was measured with an ELISA reader (Tecan, Crailsheim) at 405 nm against a reference value (492 nm). A non-linear regression standard curve was calculated (Prism software, GraphPad Inc.) using the ODs of the standard serum. After adjusting for the dilution factor relative antibody titres in relative arbitrary units (AU) were obtained.

### Stimulation of BAL cells with *P. acnes* and cytokine measurement

BAL cells were suspended in RPMI medium (Gibco, Karlsruhe, Germany) supplemented with 10 % fetal calf serum and 1 % penicillin/streptomycin and put in 24-well plates (one million BAL cells/ml/well). If indicated, BAL cells were stimulated with *P. acnes* lysate (1 μg/ml) in a 5 % CO_2_ incubator at 37 °C for 24 h. The supernatant was harvested, frozen and stored at -80 °C until cytokine measurement. TNF-α and GM-CSF were quantified in duplicates using DuoSet ELISA Development System Kits (R&D Systems Europe, UK) according to the manufacturer’s instructions.

### Statistical analysis

Values are expressed as means ± SD. Characteristics of patients with sarcoidosis and healthy volunteers were compared using a Mann-Whitney *U* test for continuous measures and Fisher’s exact test for categorical measures. Wilcoxon signed-rank test was used to compare matched samples. Spearman rank correlation was utilized to establish possible correlations between numerical values. A significance level of p < 0.05 was used in all statistics.

## Results

### Study population

The 64 patients had a mean age of 46.1 and a balanced gender distribution compared to healthy volunteers (see table 1). The healthy volunteers were significantly younger (age 28.9, p < 0.0001, table 1). 43 patients had a clinical involvement of hilar lymph nodes (radiologic stage I and II), 21 patients had not (radiologic stage III and IV). Eleven of the patients had an acute course of the disease, i.e. Löfgren syndrome. Patients with Löfgren syndrome were not significantly younger than sarcoid patients without Löfgren syndrome (age 40.9 vs. 47.1 years, *p* = 0.14).

**Table 1:** Baseline characteristics

	Healthy volunteers	Patients with sarcoidosis	*p* value
	*n* = 21	*n* = 64	
Age [years]	28.9	46.1	*p* < 0.0001
Sex [male/female]	11/10	34/30	n.s. (*p* = 1.00)
X-ray type [I/II/III/IV]	n. a.	19/24/16/5	n. a.
Löfgren syndrome [yes/no]	n. a.	11/53	n. a.
Total IgG [μg/ml] in BALF	12.4	84.3	*p* < 0.0001
Total IgA [μg/ml] in BALF	8.7	84.9	*p* < 0.0001

*Definition of abbreviations: BALF* bronchoalveolar lavage fluid, X-ray type according to Scadding [42], IgA/IgG: immunoglobulin A/G, n. a.: not applicable, n. s.: not significant

### Total IgA/IgG in BAL fluid

**Fig. 1:**
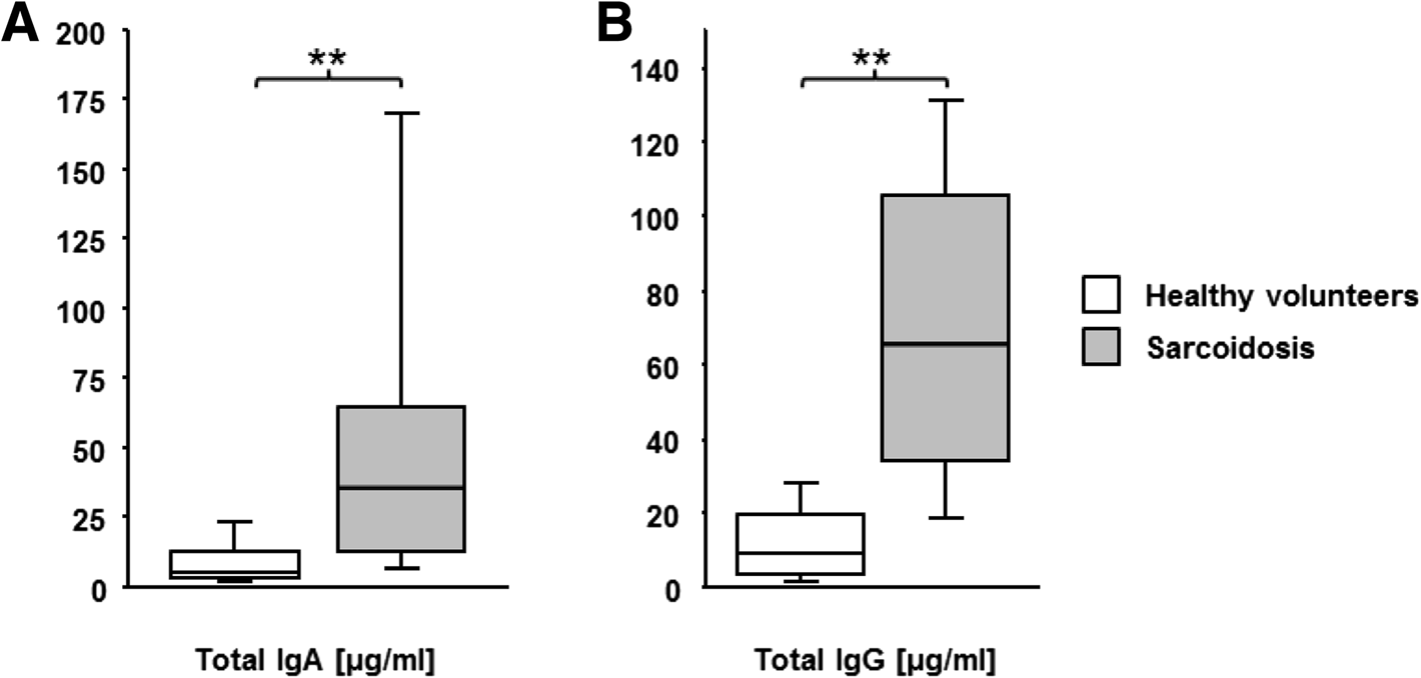
Total immunoglobulin levels in BAL fluid. Boxplots of total IgA (**a**) and total IgG (**b**) in BAL fluid of patients with sarcoidosis (grey bars) and healthy volunteers (white bars) (** *p* < 0.0001)

The BALF of patients with sarcoidosis is characterized by a significant increased amount of immunoglobulins (84.3 μg/ml IgG and 84.9 μg/ml IgA on average) compared to healthy volunteers (12.4 μg/ml IgG and 8.7 μg/ml IgA, *p* < 0.0001, Fig. 1). Patients without Löfgren syndrome or patients with radiological stage III and IV had significantly more total IgA in BALF compared with patients with Löfgren syndrome or with radiological stage I and II (*p* = 0.01 and *p* = 0.04, respectively). There was no such difference in total IgG in the BALF. There was no correlation in total IgG or total IgA and age in patients with sarcoidosis or healthy volunteers, and there was no significant difference in IgA or IgG levels comparing male and female sarcoid patients or healthy volunteers, respectively (all *p* > 0.05).

### Total specific Anti-*P. acnes*-IgG/IgA- and Anti-*S. aureus*-IgG/IgA

The total specific antibodies against *P. acnes* and against *S. aureus* were increased in patients with sarcoidosis (*p* < 0.0001). There was no difference in total IgG or IgA levels regarding patients with/without clinical involvement of hilar lymph nodes (radiological stage I/II compared to stage III/IV) or with/without Löfgren syndrome.

### Specific antibody ratio of *P. acnes* and *S. aureus*

**Fig. 2:**
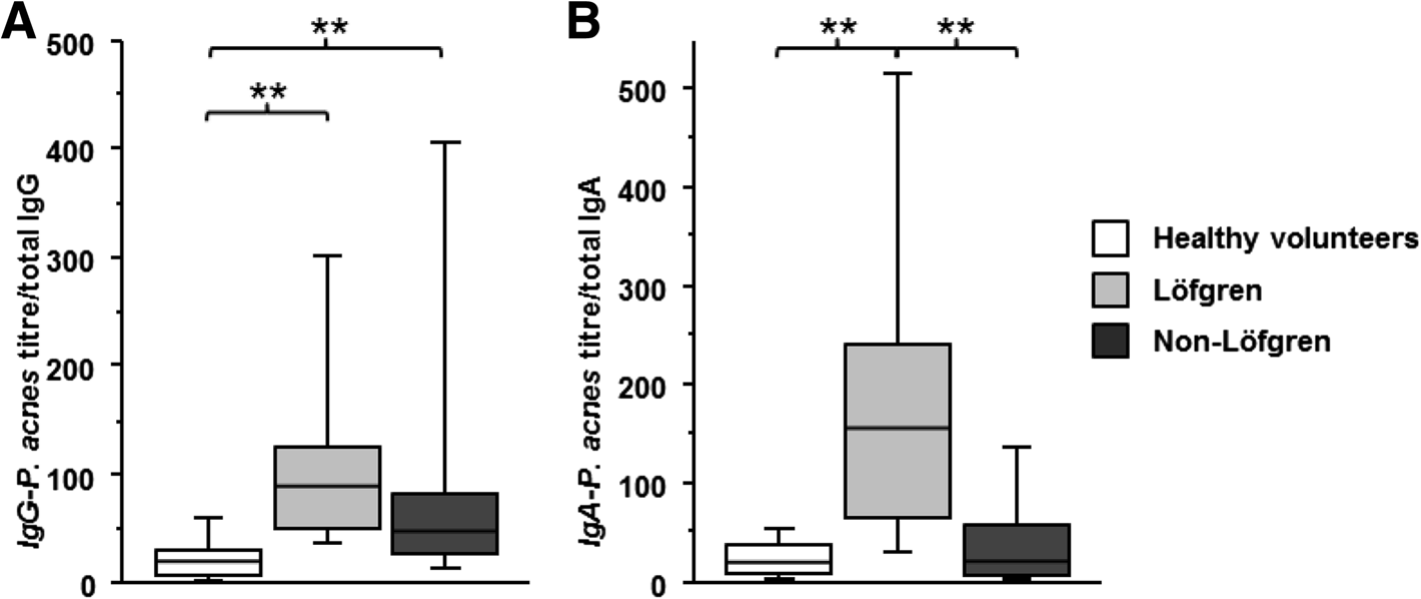
Specific antibody ratio of *P. acnes* and *S. aureus.* Boxplots of specific antibody ratios of *P. acnes* in BAL fluid of healthy volunteers (white bars), Löfgren patients (light grey bars) and non-Löfgren sarcoid patients (dark grey bars). Specific antibody ratios are expressed as ratio between Anti-*P. acnes*-IgG [arbitrary units] and total IgG [μg/ml] (**a**) or as ratio between Anti-*P. acnes*-IgA and total IgA (**b**) (**p < 0.005)

To evaluate, if there is a relative increase in specific antibodies, we calculated a ratio between the total specific anti-*P. acnes* or anti-*S. aureus* antibodies (in arbitrary units) and the total amount of immunoglobulin concentrations in the BALF. This ratio was called “specific antibody ratio”. The specific antibody ratio of *P. acnes*-IgGs were significantly increased in BALF of sarcoid patients compared to BALF of healthy volunteers (*p* = 0.0002), but not the anti-*P. acnes*-IgAs (*p* = 0.19). The anti-*P. acnes*-IgAs were significantly increased in patients with Löfgren syndrome (Fig. 2) and in patients with clinical involvement of hilar lymph nodes (radiological stage I/II). The specific antibody ratio of anti-*S. aureus*-IgG and –IgA were not increased in patients with sarcoidosis (*p* = 0.33 and p = 0.68, respectively).

### Cytokine profile of BAL cells after *P. acnes* stimulation

**Fig. 3:**
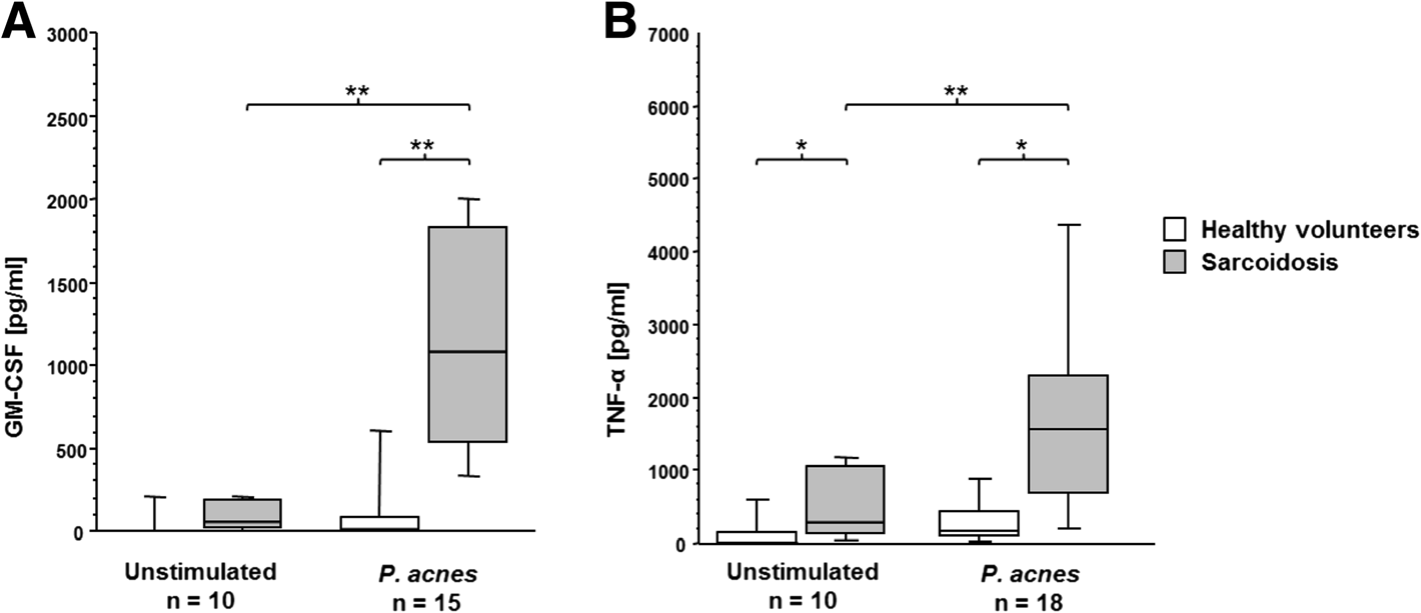
Cytokine production after P. acnes stimulation. Boxplots of GM-CSF (**a**) and TNF-α concentration (**b**) in supernatant of BAL cells from patients with sarcoidosis (*n* = 15, grey bars) and healthy volunteers (*n* = 10, white bars) that were stimulated with heat-killed *P. acnes* or left untreated (*p < 0.05, **p < 0.005)

BAL cells were cultured for 24 h with or without stimulation with heat-killed *P. acnes* (1 μg/ml). The production of the proinflammatory cytokines GM-CSF and TNF-α from untreated and *P. acnes*-stimulated BAL cells was measured by ELISA. Strikingly, unstimulated BAL cells of sarcoid patients had spontaneously elevated levels of TNF-α (530 ± 505 pg/ml vs. 145 ± 263 pg/ml, p = 0.03, Fig. 3) compared to BAL cells of healthy volunteers. *P. acnes* was neither able to significantly induce GM-CSF nor TNF-α production in BAL cells of healthy volunteers. Yet, in BAL cells from sarcoid patients, *P. acnes* strongly stimulated GM-CSF (*p* = 0.0002) and TNF-α (p = 0.001) production compared to unstimulated cells (Fig. 3). Thus, in patients with sarcoidosis, *P. acnes* stimulated BAL cells produced 1283 ± 932 pg/ml GM-CSF vs. 137 ± 270 pg/ml GM-CSF in unstimulated cells (*p* = 0.001).

## Discussion

*P. acnes* is a gram-positive, low virulent bacterium and is thought to be, beside mycobacteria, a potential factor involved in the pathogenesis of sarcoidosis. A previous study reported the presence of *P. acnes* in BAL cells and lymph nodes of patients with sarcoidosis [12]. In this study we investigated the specific immune response to *P. acnes* in sarcoid patients.

In accordance with Vandenplas and colleagues, we showed increased local immunoglobulin production, particularly higher immunoglobulin G and A concentrations in the bronchoalveolar lavage of sarcoid patients [28]. The mechanisms of hypergammaglobulinemia in sarcoidosis are not fully understood, but three points seem to be clear: First, IFNg, a hallmark cytokine in sarcoidosis, is a potent stimulus of IgG secretion by B-cells [29]. Secondly, several studies have shown, that the IgG production in sarcoidosis is compartimentalized i.e. locally produced in the lung [30, 31]. And thirdly, BAL T-cells from sarcoid patients stimulate B-cells to secret more immunoglobulins as well [30]. Furthermore, sarcoidosis is thought to be an antigen driven hypersensitivity reaction [32]. On the basis of these data, it was hypothesized that in the lungs in situ antigen presentation takes place leading to local immunoglobulin production.

Looking for specific antibodies against *P. acnes*, we used *S. aureus* as control since *S. aureus* belongs, similar as *P. acnes*, to commensals of the skin and the respiratory tract [33]. We found elevated total specific antibodies against *P. acnes* and *S. aureus* in sarcoid patients. The increased total amount of IgG and IgA in patients with sarcoidosis (Fig. 1) probably accounts for the enhanced total *S. aureus* titre, since the relative *S. aureus* titres were not elevated. This differs completely from the anti-*P. acnes* titre which are relatively and absolutely elevated (Fig. 2). Thus, the pulmonary immune system of sarcoid patients must have encountered *P. acnes* in the past. This is the first time an increased specific B-cell immune response to *P. acnes* could be proven in Caucasian sarcoid patients.

IgA and IgG prevent, amongst other things, bacteria and viruses from adhesion to the epithelium. Both, IgG and IgA are able to reach extravascular spaces; IgG by passive diffusion and IgA by an active transport across the epithelium [34]. Anti.-*P. acnes*-IgA was relatively elevated only in early disease, especially in Löfgren syndrome, while anti.-*P. acnes*-IgG was relatively increased in chronic disease as well. This finding might indicate that recent exposure to *P. acnes* is evident in Löfgren syndrome. The significance of all studies looking for a direct confirmation of *P. acnes* in sarcoid biopsies were limited to the fact that it is a commensal of the lung and also present in healthy individuals. Furthermore there is the possibility of contamination of the samples by *P. acnes*, as it is also a major commensal of the upper respiratory tract. With our approach in verifying a previously occurred exposure with *P. acnes* in vivo, we were able to abrogate this experimental limitation. Thus, our data suggest that Caucasian sarcoid patients had a recent immunologic response to *P. acnes*. Our study is limited by the fact, that our control group is significantly younger than the sarcoid patients. Aging leads to a decline of the immune system’s function, a phenomenon called “immunosenescence”; e.g., the reactivity to antigens is markedly reduced in older people [35]. We cannot exclude that part of the observed differences is due to aging, but we assume that the aging influence is marginal, because there was no correlation in sarcoid patients or healthy volunteers between total immunoglobulin levels and age. Because our study is biased regarding age, it may be possible that we underestimated the significance of relative IgG and IgA *P. acnes* titres rather than overestimated.

Next we explored, if there is an in vitro response in BAL cells stimulated with heat-killed *P. acnes*. Unstimulated BAL cells of patients with sarcoidosis already produced more TNF-α compared to control BAL cells, as shown by many groups [36, 37]. The unstimulated GM-CSF increase was not significant, possibly due to a too small sample size, and in line with Prior et. al [38]. When stimulated with heat-killed *P. acnes*, BAL cells of healthy volunteers were anergic, yet in sarcoid patients, we saw a highly significant increase in proinflammatory cytokines such as TNF-α and GM-CSF, which has never been reported before. This results suggest that stimulation with *P. acnes* leads to an exaggerated local release of cytokines necessary for granuloma and multinucleated giant cell formation, the typical histopathological changes observed in sarcoidosis [23, 24]. Of interest, increased IL-2 production and BAL T-cell proliferation following stimulation with *P. acnes* were reported by Mori and colleagues [39]. However, this phenomenon is not exclusively for sarcoidosis since also in skin acne an increase of TNF-α after *P. acnes* stimulation was reported [40]. Thus, our data indicate that contact of immune cells to *P. acnes* may boost the T_H_1 immune response in sarcoidosis.

## Conclusion

Our findings of *P. acnes* specific responses in Caucasian sarcoid patients contribute to improve the understanding of the pathogenesis of sarcoidosis. We show here that most patients with sarcoidosis have elevated levels of specific antibodies against *P. acnes* and have therefore been in contact with this bacterium in the past. Furthermore, BAL cells of sarcoid patients produce inflammatory cytokines (TNF-α and GM-CSF) upon stimulation with heat-killed *P. acnes* suggesting involvement of this pathogen in the pathogenesis of sarcoidosis. In accordance with the literature [1, 41] our study supports the notion that an unknown etiologic event disrupts the physiological tolerance towards the commensal *P. acnes* with subsequent deposition of non-degradable remnants, which gives rise to a nidus and causes granuloma formation.

## References

[1] Zissel G (2014). Cellular activation in the immune response of sarcoidosis. Semin Respir Crit Care Med.

[2] Moller DR (2007). Potential etiologic agents in sarcoidosis. Proc Am Thorac Soc.

[3] Eishi Y (2013). Etiologic aspect of sarcoidosis as an allergic endogenous infection caused by Propionibacterium acnes. Biomed Res Int.

[4] Valeyre D, Prasse A, Nunes H, Uzunhan Y, Brillet P-Y, Müller-Quernheim J (2014). Sarcoidosis. Lancet.

[5] Ishige I, Usui Y, Takemura T, Eishi Y (1999). Quantitative PCR of mycobacterial and propionibacterial DNA in lymph nodes of Japanese patients with sarcoidosis. Lancet.

[6] Chen ES, Wahlström J, Song Z, Willett MH, Wikén M, Yung RC, West EE, Mcdyer JF, Zhang Y, Eklund A, Grunewald J, Moller DR (2008). T cell responses to mycobacterial catalase-peroxidase profile a pathogenic antigen in systemic sarcoidosis. J Immunol.

[7] Nakata Y, Kataoka M, Ejiri T, Mori Y, Hioka T, Maeda T, Hosoya S, Ohnoshi T, Kimura I (1989). The response of alveolar lymphocytes induced by Propionibacterium acnes in pulmonary sarcoidosis: correlation with clinical studies, pulmonary function studies and bronchoalveolar lavage. Nihon Kyobu Shikkan Gakkai Zasshi.

[8] Carlisle J, Evans W, Hajizadeh R, Nadaf M, Shepherd B, Ott RD, Richter K, Drake W (2007). Multiple Mycobacterium antigens induce interferon-gamma production from sarcoidosis peripheral blood mononuclear cells. Clin Exp Immunol.

[9] Orme IM, Andersen P, Boom WH (1993). T cell response to Mycobacterium tuberculosis. J Infect Dis.

[10] Ishige I, Eishi Y, Takemura T, Kobayashi I, Nakata K, Tanaka I, Nagaoka S, Iwai K, Watanabe K, Takizawa T, Koike M (2005). Propionibacterium acnes is the most common bacterium commensal in peripheral lung tissue and mediastinal lymph nodes from subjects without sarcoidosis. Sarcoidosis Vasc Diffuse Lung Dis.

[11] Scott MT, Milas L (1977). The distribution and persistence in vivo of Corynebacterium parvum in relation to its antitumor activity. Cancer Res.

[12] Homma JY, Abe C, Chosa H, Ueda K, Saegusa J, Nakayama M, Homma H, Washizaki M, Okano H (1978). Bacteriological investigation on biopsy specimens from patients with sarcoidosis. Jpn J Exp Med.

[13] Hiramatsu J, Kataoka M, Nakata Y, Okazaki K, Tada S, Tanimoto M, Eishi Y (2003). Propionibacterium acnes DNA detected in bronchoalveolar lavage cells from patients with sarcoidosis. Sarcoidosis Vasc Diffuse Lung Dis.

[14] Ichikawa H, Kataoka M, Hiramatsu J, Ohmori M, Tanimoto Y, Kanehiro A, Nakata Y, Tanimoto M (2008). Quantitative analysis of propionibacterial DNA in bronchoalveolar lavage cells from patients with sarcoidosis. Sarcoidosis Vasc Diffuse Lung Dis.

[15] Negi M, Takemura T, Guzman J, Uchida K, Furukawa A, Suzuki Y, Iida T, Ishige I, Minami J, Yamada T, Kawachi H, Costabel U, Eishi Y (2012). Localization of propionibacterium acnes in granulomas supports a possible etiologic link between sarcoidosis and the bacterium. Mod Pathol.

[16] Eishi Y, Suga M, Ishige I, Kobayashi D, Yamada T, Takemura T, Takizawa T, Koike M, Kudoh S, Costabel U, Guzman J, Rizzato G, Gambacorta M, du Bois R, Nicholson AG, Sharma OP, Ando M (2002). Quantitative analysis of mycobacterial and propionibacterial DNA in lymph nodes of Japanese and European patients with sarcoidosis. J Clin Microbiol.

[17] Ebe Y, Ikushima S, Yamaguchi T, Kohno K, Azuma A, Sato K, Ishige I, Usui Y, Takemura T, Eishi Y (2000). Proliferative response of peripheral blood mononuclear cells and levels of antibody to recombinant protein from Propionibacterium acnes DNA expression library in Japanese patients with sarcoidosis. Sarcoidosis Vasc Diffuse Lung Dis.

[18] Nishiwaki T, Yoneyama H, Eishi Y, Matsuo N, Tatsumi K, Kimura H, Kuriyama T, Matsushima K (2004). Indigenous pulmonary Propionibacterium acnes primes the host in the development of sarcoid-like pulmonary granulomatosis in mice. Am J Pathol.

[19] McCaskill JG, Chason KD, Hua X, Neuringer IP, Ghio AJ, Funkhouser WK, Tilley SL (2006). Pulmonary immune responses to Propionibacterium acnes in C57BL/6 and BALB/c mice. Am J Respir Cell Mol Biol.

[20] Iio K, Iio TU, Okui Y, Ichikawa H, Tanimoto Y, Miyahara N, Kanehiro A, Tanimoto M, Nakata Y, Kataoka M (2010). Experimental pulmonary granuloma mimicking sarcoidosis induced by Propionibacterium acnes in mice. Acta Med Okayama.

[21] Tchaptchet S, Gumenscheimer M, Kalis C, Freudenberg N, Hölscher C, Kirschning CJ, Lamers M, Galanos C, Freudenberg MA (2012). TLR9-dependent and independent pathways drive activation of the immune system by Propionibacterium acnes. PLoS One.

[22] Ziegenhagen MW, Schrum S, Zissel G, Zipfel PF, Schlaak M, Müller-Quernheim J (1998). Increased expression of proinflammatory chemokines in bronchoalveolar lavage cells of patients with progressing idiopathic pulmonary fibrosis and sarcoidosis. J Investig Med.

[23] Smith D, Hänsch H, Bancroft G, Ehlers S (1997). T-cell-independent granuloma formation in response to Mycobacterium avium: role of tumour necrosis factor-alpha and interferon-gamma. Immunology.

[24] Broos CE, van Nimwegen M, Hoogsteden HC, Hendriks RW, Kool M, van den Blink B (2013). Granuloma Formation in Pulmonary Sarcoidosis. Front Immunol.

[25] Lemaire I, Yang H, Lauzon W, Gendron N (1996). M-CSF and GM-CSF promote alveolar macrophage differentiation into multinucleated giant cells with distinct phenotypes. J Leukoc Biol.

[26] Prasse A, Pechkovsky DV, Toews GB, Jungraithmayr W, Kollert F, Goldmann T, Vollmer E, Müller-Quernheim J, Zissel G (2006). A vicious circle of alveolar macrophages and fibroblasts perpetuates pulmonary fibrosis via CCL18. Am J Respir Crit Care Med.

[27] Sing A, Merlin T, Knopf HP, Nielsen PJ, Loppnow H, Galanos C, Freudenberg MA (2000). Bacterial induction of beta interferon in mice is a function of the lipopolysaccharide component. Infect Immun.

[28] Vandenplas O, Depelchin S, Delaunois L, Delwiche JP, Sibille Y (1994). Bronchoalveolar lavage immunoglobulin A and G and antiproteases correlate with changes in diffusion indices during the natural course of pulmonary sarcoidosis. Eur Respir J.

[29] Snapper CM, Rosas F, Moorman MA, Jin L, Shanebeck K, Klinman DM, Kehry MR, Mond JJ, Maliszewski CR (1996). IFN-gamma is a potent inducer of Ig secretion by sort-purified murine B cells activated through the mIg, but not the CD40, signaling pathway. Int Immunol.

[30] Hunninghake GW, Crystal RG (1981). Mechanisms of hypergammaglobulinemia in pulmonary sarcoidosis. Site of increased antibody production and role of T lymphocytes. J Clin Invest.

[31] Rankin JA, Naegel GP, Schrader CE, Matthay RA, Reynolds HY (1983). Air-space immunoglobulin production and levels in bronchoalveolar lavage fluid of normal subjects and patients with sarcoidosis. Am Rev Respir Dis.

[32] Grunewald J, Eklund A, Wigzell H, Van Meijgaarden KE, Ottenhoff TH (1999). Bronchoalveolar lavage cells from sarcoidosis patients and healthy controls can efficiently present antigens. J Intern Med.

[33] Robinson J (2004). Colonization and infection of the respiratory tract: What do we know?. Paediatr Child Health.

[34] Janeway CJ, Travers P, Walport M, Shlomchik M: The distribution and functions of immunoglobulin isotypes. In Immunobiology: The Immune System in Health and Disease. 5th edition. Garland Science; 2001.

[35] Goronzy JJ, Weyand CM (2013). Understanding immunosenescence to improve responses to vaccines. Nat Immunol.

[36] Müller-Quernheim J, Pfeifer S, Männel D, Strausz J, Ferlinz R (1992). Lung-restricted activation of the alveolar macrophage/monocyte system in pulmonary sarcoidosis. Am Rev Respir Dis.

[37] Baughman RP, Strohofer SA, Buchsbaum J, Lower EE (1990). Release of tumor necrosis factor by alveolar macrophages of patients with sarcoidosis. J Lab Clin Med.

[38] Prior C, Knight RA, Herold M, Ott G, Spiteri MA (1996). Pulmonary sarcoidosis: patterns of cytokine release in vitro. Eur Respir J.

[39] Mori Y, Nakata Y, Kataoka M, Ejiri T, Hioka T, Maeda T, Hosoya S, Ohnoshi T, Kimura I (1989). Interleukin-2 production and receptor expression of alveolar lymphocytes stimulated by Propionibacterium acnes in sarcoidosis. Nihon Kyobu Shikkan Gakkai Zasshi.

[40] Jasson F, Nagy I, Knol AC, Zuliani T, Khammari A, Dréno B (2013). Different strains of Propionibacterium acnes modulate differently the cutaneous innate immunity. Exp Dermatol.

[41] Chen ES, Moller DR (2014). Etiologic role of infectious agents. Semin Respir Crit Care Med.

[42] Scadding JG (1961). Prognosis of intrathoracic sarcoidosis in England. A review of 136 cases after five years’ observation. Br Med J.

